# Analysis on Soil Seed Bank Diversity Characteristics and Its Relation with Soil Physical and Chemical Properties after Substrate Addition

**DOI:** 10.1371/journal.pone.0147439

**Published:** 2016-01-25

**Authors:** Mengxuan He, Lingyue Lv, Hongyuan Li, Weiqing Meng, Na Zhao

**Affiliations:** 1 College of Environmental Science and Engineering, Nankai University, Tianjin, China; 2 College of Urban and Environment Science, Tianjin Normal University, Tianjin, China; Henan Agricultural Univerisity, CHINA

## Abstract

**Aims:**

Considered as an essential measure in the application of soil seed bank (SSB) projects, the mixing of substrate and surface soil can effectively improve soil condition. This research is aimed at exploring the diversity characteristics of SSBs and the relationships between SSBs and soil properties.

**Methods:**

Canonical correspondence analysis (CCA) was adopted to describe the ordination of SSBs on soil properties’ gradients; multiple linear regressions were adopted to analyze the relationship between average growth height and soil properties, density and soil properties.

**Results:**

Experimental groups of mixed substrate (the mixture of organic and inorganic substrates) had high diversity indexes, especially the Shannon-Wiener Index compared with those of single substrate. Meanwhile, a higher number of species and increased density were also noted in those of mixed substrate. The best test group, No.16, had the highest diversity indexes with a Shannon-Wiener of 1.898, Simpson of 0.633 and Pielou of 0.717, and also showed the highest density of 14000 germinants /m^2^ and 21 species. In addition, an improvement of the soil’s chemical and physical properties was noted when the substrates were mixed. The mixed substrate of turfy soil and perlite could effectively enhance the soil moisture content, whilst a mixed substrate of rice husk carbon and vermiculite could improve the content of available potassium (AK) and phosphorus (AP) and strengthen soil fertility. The germinated plants also reflected obvious regularities of ordination on soil factor gradients. Three distinct cluster groups were presented, of which the first cluster was distributed in an area with a relatively higher content of AK and AP; the second cluster was distributed at places with relatively higher soil moisture content; and the third cluster of plants didn’t show any obvious relationship with soil physical and chemical properties. Through CCA analysis, AK and AP were considered the most important soil factors to influence the SSB, which was illustrated in regression analysis with a high correlation coefficient when dependent values were growth height and density respectively. The linear regression equations with: growth height = 142.728 − 1.514TC + 30.218AP − 5.083TN + 10.839AK + 1.234mc; density = −68.216 − 4.609TC + 9.566AP − 35.492TN + 19.779AK − 1.591mc.were established by multiple linear regression.

**Conclusions:**

(1) The mixed substrates showed a greater advantage for SSBs than single substrates, both in improving the number of species, density and diversity indexes. (2) The germinated plants reflected an obvious preference to different soil conditions, which different mixed substrates could improve accordingly. (3) AK, AP were important soil factors to influence SSBs, especially in the growth of plants and density.

## Introduction

A soil seed bank is a collection of viable seeds present in the soil with regeneration potential [1, 2]. It has been demonstrated a SSB possesses the potential vegetation recovery capacity [3,4] and the importance of SSBs were discovered in many ecosystems and documented with information crucial for understanding vegetation dynamics [5,6]. Compared with other vegetation recovery methods, a SSB has the advantage in maintaining the balance of the local ecological system, especially in protecting local native species [7–9].

Successful projects using SSBs for landscape engineering in slopes or dams were mainly carried out in Japan [10–12] and this method was called the “Surface Soil Greening Method” [13]. Some typical engineering cases examined were: The topsoil spraying for the Mino River Dam natural recovery [14]; the revegetation method using topsoil seed banks in Yakushima Island [15]. In all engineering projects, including the above two, the method of spraying the mixture of surface soil and substrate was adopted more often than spraying surface soil directly. The use of substrate will not only reduce the transplantation volume of surface soil to save project cost [16], but also increase the effect of providing plant roots with water, fertilizer and space. High-quality substrate is therefore considered essential for the growth of plants [17, 18].

However in China, the focuses on SSB are mainly about the fundamental study [19–22] without any practical application projects, and studies about substrate and surface soil are blank. This research was inspired by the “Surface Soil Greening Method” of Japan, and aimed to find the effects on SSBs of using different substrates. The purpose of this paper could be divided into three aspects: 1、exploring the effect on SSB diversity indexes by adding different substrates 2、researching the ordination rules of SSBs on soil factor gradients after mixing substrate through CCA 3、exploring the optimal substrate solution to improve soil properties and finding the most important factors that affected SSBs. Although this simulation of germination tests was carried out in a greenhouse, it was still an innovation to explore engineering application measures which could provide valuable reference for SSBs engineering projects in the future.

## Materials and Methods

### Surface soil sampling site and Substrates material

Of all its numerous reserves of SSBs compiled by earlier researchers [23], surface soil collection sites were selected in the Wuqing District of Tianjin, in an abandoned sparse woodland area with many herbaceous plants and where sampling was allowed and legal without any endangered or protected species. In March 2014, 10 random quadrats with an area of 1m × 1m were selected in the site, and five sampling points with the area of 30 cm × 30 cm were selected in the center, east, west, south, and north and were arranged at an interval of 1 m. These sampling points were all of a 20 cm depth from the ground surface. Distinct roots and gravel were manually removed and samples were filtered by a fine sieve with size of 80 mesh per inch [24, 25]. Concentrated samples were sealed separately into valve bags for germination tests.

In the practical seedling applications, such as agriculture、horticulture and flower cultivation, the use of substrate is a very common phenomenon [26–28]. Classified by components of the substrate, three common types were divided including: organic substrate, inorganic substrate and mixed substrate [29–32]. In this research, followed by the classification in the seedling area, these three types of substrate were all selected, in which organic substrates included turfy soil and rice husk carbon; inorganic substrates included activated carbon and vermiculite; mixed substrates were the mixture of turfy soil and perlite (volume ratio was 2:1 and 1:1 respectively) and the mixture of rice husk carbon and vermiculite (volume ratio 2:1 and 1:1 respectively).The reason for choosing these substrates were that these substrates had followed the principle that they were abundant resources in Tianjin, widely used and easily obtained in Tianjin, and with a low price. For pre-treatment, the substrates and surface soil were mixed evenly by a shovel in a flat place on a plastic membrane after calculating its volume and then were put back into the nursery trays.

### The experimental schemes

Sixteen experimental groups, which were groups with the best restoration effect covering organic substrates、inorganic substrates and mixed substrates selected from 44 experimental groups in my master thesis [33], were analyzed. The analysis of these 16 groups with relatively high diversification levels and densities will be more accurate and significant in the actual application project. The detail groups were shown in Table 1.

**Table 1:** The experimental groups of SSB germination.

Testing No.	Name of substrate	Ratio of mixing with surface soil (volume ratio)
1	rice husk carbon	30%
2	rice husk carbon	40%
3	turfy soil	30%
4	turfy soil	40%
5	activated carbon	10%
6	activated carbon	20%
7	vermiculite	10%
8	vermiculite	20%
9	turfy soil and perlite 2:1(volume ratio)	20%
10	turfy soil and perlite 1:1(volume ratio)	20%
11	turfy soil and perlite 2:1(volume ratio)	30%
12	turfy soil and perlite 1:1(volume ratio)	30%
13	rice husk carbon and vermiculite2:1 (volume ratio)	20%
14	rice husk carbon and vermiculite 1:1 (volume ratio)	20%
15	rice husk carbon and vermiculite 2:1 (volume ratio)	30%
16	rice husk carbon and vermiculite 1:1 (volume ratio)	30%

### Germination experiments

The germination greenhouse was located at the Tianjin University of Technology, where germination experiments were implemented. The soil in the sampling area is compacted soil, so after removal of gravel or dead leaves, soil samples were crushed by hammer then mixed evenly with different substrates based on the mixing volume ratio (Table 1). The mixture of substrate and surface soil were laid in the trays to a thickness of 5cm. The germination time lasted about three months from Mar 5, 2014 to May 30, 2014, during which time the soil was watered daily to maintain the soil moisture and the characteristics of the SSBs were recorded regularly. The germination of SSBs was in process until all the species germinated without any new emergences. The analysis data was determined a month later when plant communities reached a state without any new species emerging. The number of seedlings, number of species, and the height of every species, its coverage and biomass were recorded.

The average growth height of the community, which could approximately represent the canopy height, was calculated by adding the average height of per different species and then dividing it by the number of species.

### Test of soil physical and chemical properties

Because all 16 experimental groups contained added substrates, the soil conditions could be effectively improved. Five soil factors were measured (S1 Table), including: moisture content (mc), available potassium (AK), available phosphorus (AP), total carbon (TC) and total nitrogen (TN). The apparatuses used in soil pretreatment were a TS-100B constant temperature shaking incubator, a sieve with 60 meshes; The moisture content was measured by the electro-thermal blowing dry oven and electronic balance by AL104; available phosphorus was measured by ICP-OES; available potassium was measured by TAS-990 atomic absorption spectrophotometer; and total carbon and total nitrogen were measured by Elementa Vario EL III CHNO analyzer.

### Data analyzing methods

The diversity characteristics of SSB were calculated using R software and the graphs were drawn by Excel. For exploring the ordination between SSB and soil physical and chemical properties, CCA was adopted, which is a new method generated from modification of CA/RA [34–36] with each step of calculation results regressing with environmental factors [37].CCA analysis was completed using Canoco for Windows 4.5 software.

Quantitative analysis was made on the soil’s physical and chemical properties and the community features, including average community height and density by the linear multivariate regression method, which can jointly evaluate dependent variables through the optimal combination of multiple independent variables [38]. This analysis was completed in SPSS software.

## Results

### The diversity indexes of SSB after different substrates addition

In order to better understand the diversity indexes, the details of species per experimental group were shown in Table 2. The density and number of species differed depending on the addition of different substrates. Density was obviously higher in groups with mixed substrate (including organic and inorganic substrate) than those of single substrate. Although *Setaria viridis*、*Digitaria sanguinalis*、*Cyperus compressus* were the dominant species with high density per group, the densities of these species are far higher in the groups of mixed substrates. In addition, the germination of *Chenopodium glaucum*、*Sonchus wightianus*、*Amaranthus cruentus* and *Chenopodium album* contributed a lot to enhance the density and number of species.

**Table 2:** The germination condition in per experimental group (germinants. m^-2^).

Species	1	2	3	4	5	6	7	8	9	10	11	12	13	14	15	16
*Setaria viridis*	3140	2140	3360	2100	2820	2440	3200	5060	3700	3920	3320	5400	3380	3860	3800	4220
*Mulgedium tataricum*	20	80	20	40	20	60	20	80	100	80	40	240	320	100	120	220
*Eclipta prostrata*	60	20	60	20	20	100	20	20	60	60	80	120	320	700	20	140
*Digitaria sanguinalis*	1960	2580	660	820	1240	920	1120	820	1980	3660	2780	1320	2180	2440	2480	3720
*Portulaca oleracea*	40	20	20	20	80	80	100	160	20	220	240	240	60	160	160	80
*Lactuca indica*	100	60	20	320	20	200	40	120	20	40	40	200	520	460	20	180
*Youngia japonica*	40	60	80	20	20	20	40	100	20	160	120	180	100	80	40	200
*Cyperus compressus*	480	440	380	300	600	440	480	440	600	560	460	500	260	280	200	280
*Ixeridium sonchifolium*	20	20	20	0	20	20	60	40	100	60	0	100	420	40	680	80
*Viola prionantha*	40	20	0	0	120	80	40	40	40	40	80	100	680	20	20	20
*Amaranthus cruentus*	200	300	0	0	0	420	300	460	300	420	300	100	60	20	20	820
*Chenopodium album*	100	40	0	0	0	200	100	20	40	180	40	80	140	420	180	360
*Convolvulus arvensis*	60	20	0	0	0	0	100	120	40	20	0	80	40	340	80	40
*Chenopodium glaucum*	300	660	0	0	0	0	300	20	40	180	80	40	40	0	20	1480
*Sonchus wightianus*	100	0	0	0	0	0	20	20	80	200	120	40	1820	20	600	1440
*Cirsium arvense var*. *integrifolium*	0	0	0	0	0	0	20	20	0	20	160	40	0	0	0	20
*Abutilon theophrasti*	0	0	0	0	0	0	60	20	0	100	80	40	1580	0	0	160
*Ailanthus altissima*	0	0	0	0	0	0	0	0	0	60	0	40	0	0	0	40
*Pharbitis nil*	0	0	0	0	0	0	40	60	0	20	0	20	480	400	200	460
*Koelreuteria paniculata*	0	0	0	0	0	0	0	0	0	20	0	20	0	0	0	20
*Fraxinus chinensis*	0	0	0	0	0	0	0	0	0	0	0	0	0	0	0	20
total	6660	6460	4620	3640	4960	4980	6060	7620	7140	10920	7940	8900	12400	9340	8640	14000

The highest density was 14000 germinants/m^2^ with 21 species in Testing No.16, namely the mixed substrate composed of rice husk carbon and vermiculite with the volume ratio of 1:1 when mixed again with surface soil at the ratio of 30%. The lowest group was Testing No.4 with its density of 3640 germinants/m^2^ and only 8 species emerged. This lowest group had the addition of turfy soil, a kind of organic substrate.

**Fig 1:**
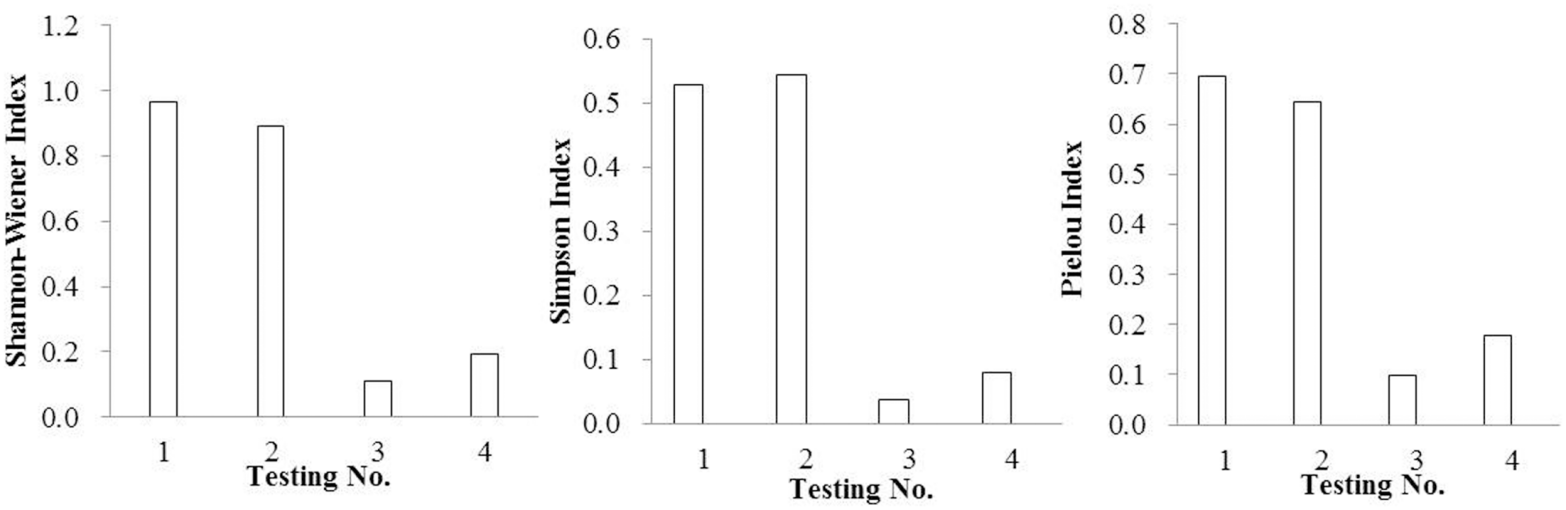
The diversity indexes of SSB by adding organic substrates.

**Fig 2:**
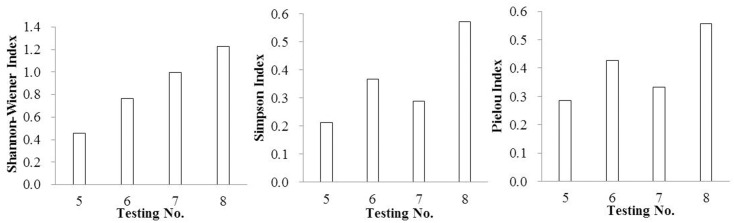
The diversity indexes of SSB by adding inorganic substrates.

**Fig 3:**
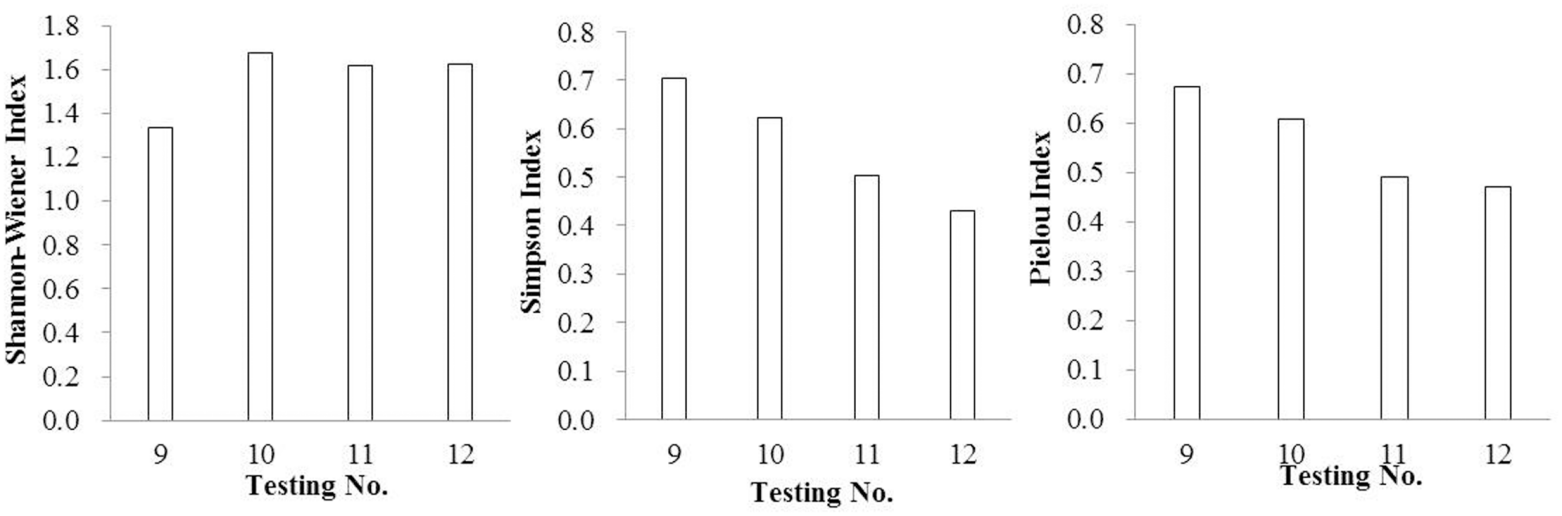
The diversity indexes of SSB by using mixed substrate (including turfy soil and perlite).

**Fig 4:**
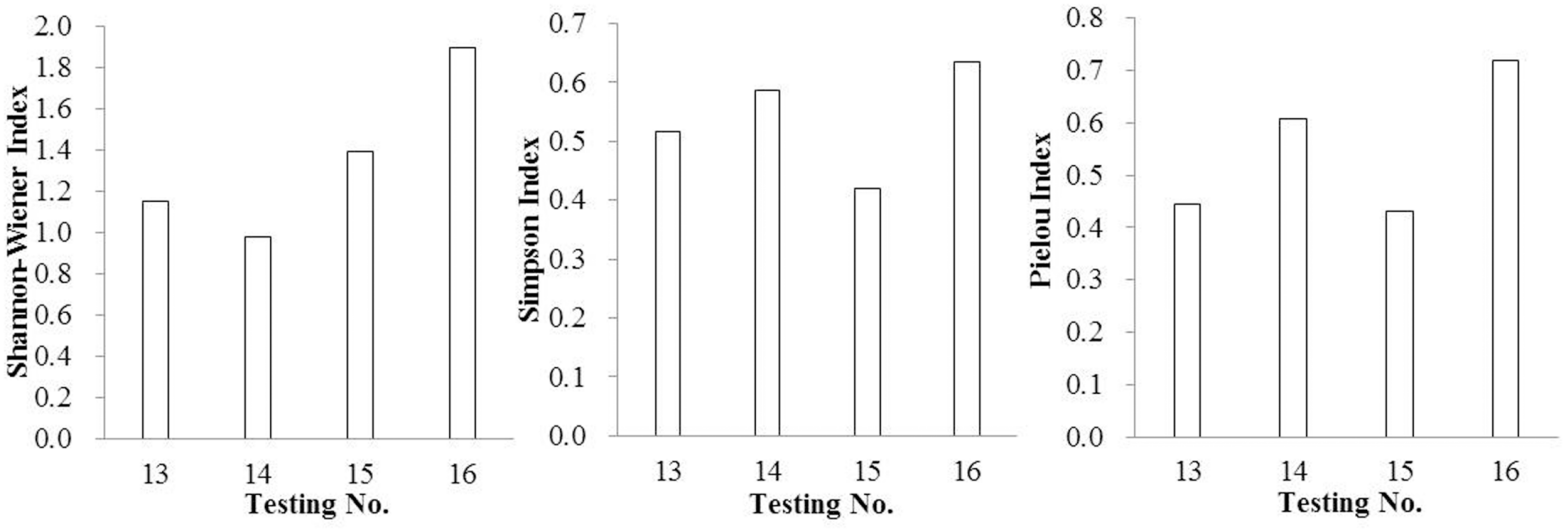
The diversity indexes of SSB by using mixed substrate (including rice husk carbon and vermiculite).

Species diversity indexes are important indexes to reflect the stability of the community and the complexity function of the community [39–42]. Higher species diversities always indicate better ecological restoration effects [43–46]. In this study, diversity indexes were reflected in Figs 1–4.

In reference to organic substrate, the Shannon-Wiener Index, Pielou Index and Simpson Index were significantly improved by mixing rice husk carbon rather than turfy soil, especially at the ratio of 30%. In the case of inorganic substrate, vermiculite showed distinct advantages with the mixing ratio of 20%.

As a whole, the effect of improving community diversity indexes showed advantage in mixed substrate rather than single substrate, especially the Shannon-Wiener Index, which had the same regularity as density and number of species. Taking the Shannon-Wiener Index、Pielou Index and Simpson Index into consideration, the mixed substrate composed of rice husk carbon and vermiculite with the volume ratio 1:1 was the most effective when mixed again with surface soil at the ratio of 30%. Also noteworthy was the fact that in this group the density of *Setaria viridis*、*Digitaria sanguinalis*、*Cyperus compressus*、 *Chenopodium glaucum*、*Sonchus wightianus*、*Amaranthus cruentus*、*Chenopodium album* were very high with 4220、3720、280、1480、1440、820 and 360 germinants/m^2^ respectively.

The tendency of the Simpson Index and Pielou index were almost the same when the substrate changed, which was very different from Shannon-Wiener Index.

### CCA analysis of plant species and soil physical and chemical properties

CCA ordination could well reflect both the distribution of species on environmental factor gradients and the distribution of quadrats (one tray was considered as a quadrat) on environmental factor gradients [47–49].The statistical results of CCA were shown in Table 3. Cumulative percentage variance of species data for the first four axes reached up to 37.8%, which indicated other factors could also influence the distribution of species in this biplot as well as soil properties [50–51].

**Table 3:** Statistic Results of CCA Ordination.

Axes	1	2	3	4
Eigenvalues	0.353	0.142	0.030	0.017
Species-environment correlations	0.779	0.644	0.556	0.484
Cumulative percentage variance of species data	24.7	34.6	36.6	37.8
Cumulative percentage variance of species-environment relation	63.7	89.2	94.6	97.6

The eigenvalues of the first and second ordination axes were 0.353 and 0.142, which were higher than the characteristic values of the third and fourth ordination axes, and species-environment correlations on the first and second axes were obviously higher than that of the third and fourth axes. The cumulative percentage variances of the species-environment relationship on the first two ordination axes were as high as 89.2%. Therefore, the information contained in the first two ordination axes could represent most of the information.

**Fig 5:**
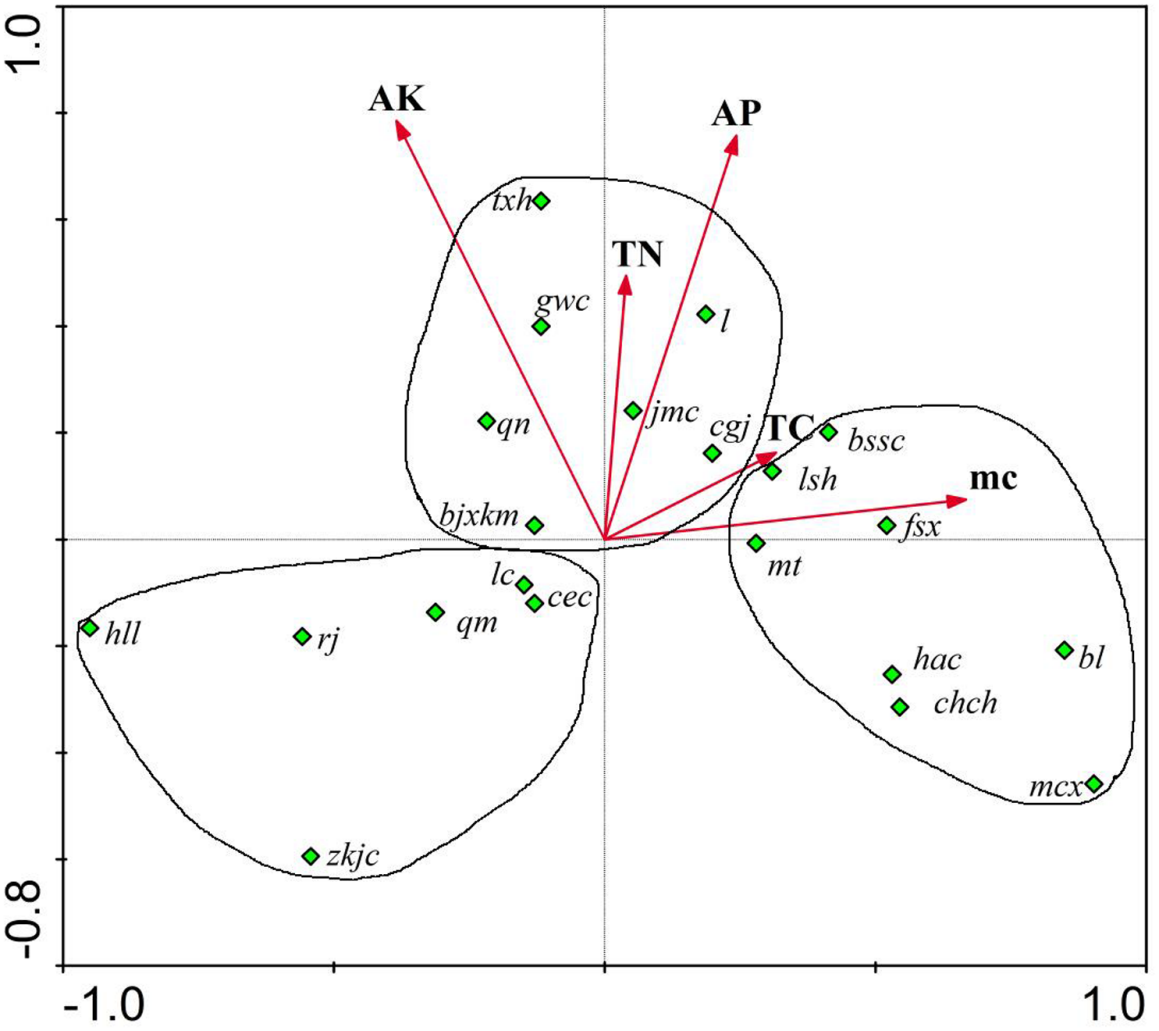
CCA ordination program between species and soil properties. **Annotation: txh**-*Convolvulus arvensis*; **gwc**-*Setaria viridis*; **l**- *Chenopodium album*; **jmc**- *Sonchus wightianus*; **cgj**- *Lactuca indica*; **qn**- *Pharbitis nil*; **bjxkm**- *Ixeridium sonchifolium*; **bssc**- *Cyperus compressus*; **lsh**- *Koelreuteria paniculata*; **fsx**- *Amaranthus cruentus*; **mt**- *Digitaria sanguinalis*; **hac**-*Youngia japonica*; **chch**- *Ailanthus altissima*; **bl**-*Fraxinus chinensis*; **mcx**- *Portulaca oleracea*; **lc**- *Eclipta prostrata*; **cec**- *Cirsium arvense var*. *integrifolium*; **qm**- *Abutilon theophrasti*; **rj**- *Mulgedium tataricum*; **hll**- *Chenopodium glaucum*; **zkjc**- *Viola prionantha*.

The two-dimensional ordination diagram reflecting the relationship of germinated plant species and physical and soil chemical properties was drawn based on the first two ordination axes (Fig 5).

The plant cluster regulation could authentically reflect the growth habit of plants and the preference to soil nature, demonstrating that the soil properties will influence plants. As shown in Fig 5, most species of the SSB were distributed around the soil factors and three obvious clusters were shown based on different soil factor gradients, of which the first cluster was distributed around the area with a relatively high content of AK and AP. The plants mainly included *Convolvulus arvensis*、*Setaria viridis*、*Sonchus wightianus*、*Chenopodium album*、*Pharbitis nil*, etc. The second cluster was distributed around the area with high soil moisture content. The plants mainly included *Koelreuteria paniculata*、*Amaranthus cruentus*、*Digitaria sanguinalis*、*Ailanthus altissima*、*Fraxinus chinensis*、*Portulaca oleracea*,etc. The third cluster was distributed in the direction converse to the arrow. The plants in this area mainly included *Viola prionantha*、*Chenopodium glaucum*、*Mulgedium tataricum*、*Abutilon theophrasti*、*Cirsium arvense var*. *integrifolium*,etc.

The regulation of plant clusters can reflect plants’ preference of soil, which has an important instructive significance for engineering projects to improve soil nature and promote growth of plants.

### CCA analysis of quadrats and soil physical and chemical properties

**Fig 6:**
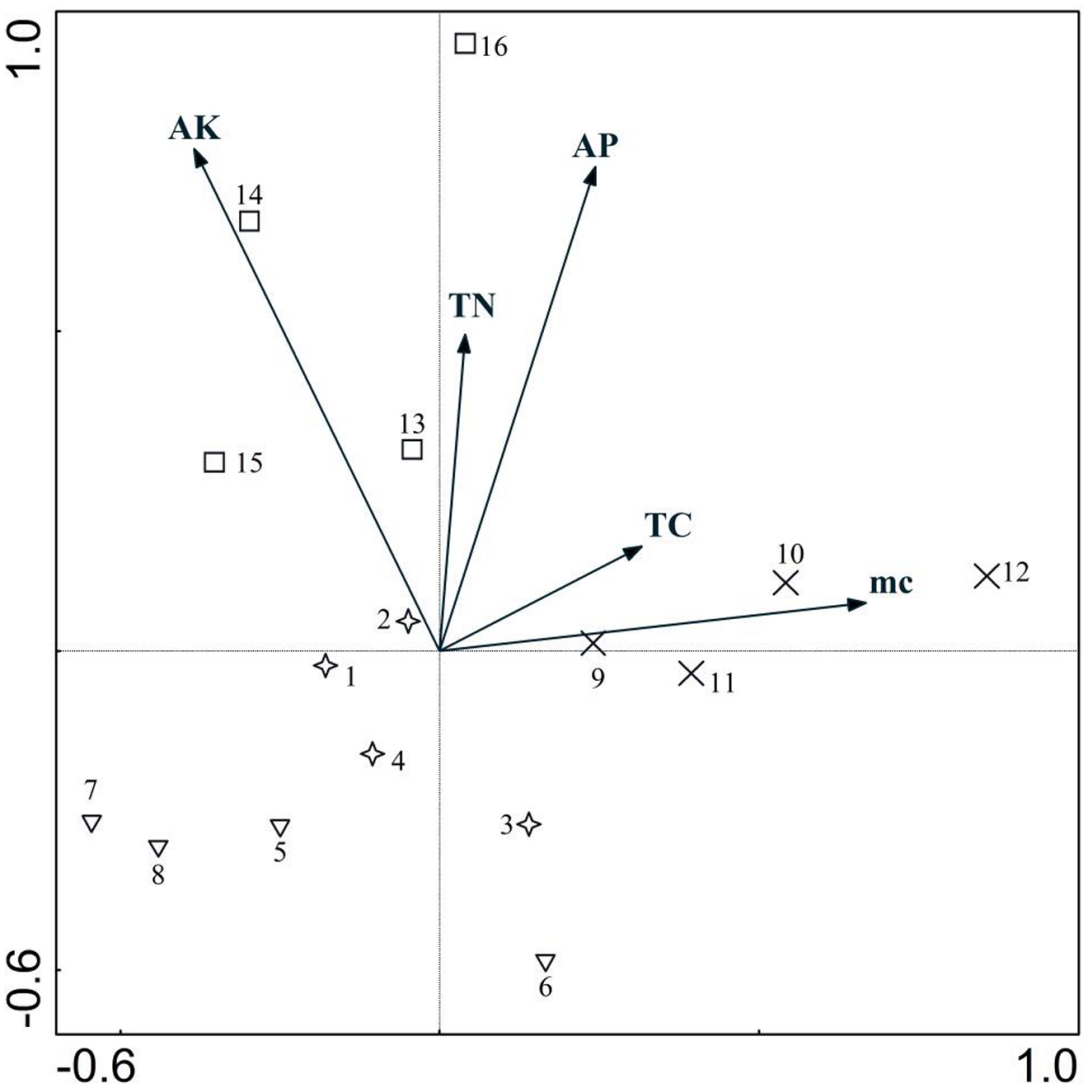
CCA ordination program between quadrats and soil properties.

Therefore to clearly illustrate the influence on soil by using different substrates, another two-dimensional ordination diagram was drawn (Fig 6), where the serial numbers were consistent with those in Table 1.

Different quadrats (experimental groups) have clustered around different soil factors, indicating that different substrates had exerted different effects on soil properties. Groups with single substrates distributed in the area far from soil factor gradients, which could be seen from the bottom left of the diagram, whilst those distributed around soil factors gradients were all experiment groups with mixed substrate, which revealed the important influence in improving the soil properties compared with single substrate. The same conclusion could also be drawn when referring to diversity indexes.

Those distributed around AK and AP were mixed substrates composed of rice husk carbon and vermiculite, so this mixed substrate could well improve the content of soil organic elements such as K、P, especially when the mixture ratio with surface soil was 30%. Those distributed in the area with relatively high moisture content were of mixed substrates composed of turfy soil and perlite, which indicated this kind of mixed substrate was relatively poor in providing organic elements, but it had great advantage in maintaining soil moisture, and the effect would also be the best if it was mixed with surface soil at the ratio of 30%.

In the CCA ordination diagram, the arrow represented soil physical and chemical factors and its length indicated the correlation between plants and the environmental factors [52–54]. It could be concluded from the diagram that both AK and AP were very important soil factors for SSBs. AP and AK were both microelements needed by plants, which could promote the fertility of soil and be well absorbed by plants. They are essential elements for the growth of the whole community [55, 56]. Although the moisture content of soil was less important than the above two factors, it still couldn’t be ignored. Soil had a high requirement for water content for plant roots, so the moisture content was also not a negligible factor [57].

### Relations between community features and soil physical and chemical properties

The average growth height (a) and density (germinants/dm^2^) (b) were chosen to explore the relationship with soil physical and chemical properties by using linear multivariate regression, providing important reference for the promotion of plant growth and its density. The linear multivariate regression analysis method is useful, for it can form relationship equations and is more quantitative and intuitive. The results of ANOVA analysis were shown in Tables 4 and 5.

**Table 4:** ANOVA results of the linear multivariate regression analysis (a).

Model	Sum of Squares	df	Mean square	F	Sig.
Regression	464.978	5	92.996	5.480	0.011
Residual	169.698	10	16.970		
Total	634.677	15			

**Table 5:** ANOVA results of the linear multivariate regression analysis (b).

Model	Sum of Squares	df	Mean square	F	Sig.
Regression	9764.744	5	1952.949	7.510	0.004
Residual	2600.366	10	260.037		
Total	12365.110	15			

For the growth height, the sum of squares for Regression was 464.978 and this model had a good statistical significance with an F-value of 5.480 and p = 0.011, which demonstrated the model was very significant. In case of density, its sum of squares was 9764.744 and it also had a very significant model with its p = 0.004.

Meanwhile the adjusted R^2^ were 0.733 and 0.685,which were very close to 1, illustrating the two models adequately fit the data again [58], so the two regression equations could explain clearly the relationship between soil properties and average growth height and the relationship between soil properties and density.

As shown in Table 6, AP and AK had great influence on the average growth height with higher correlation coefficients than other factors. AP and AK also had good statistical significance with both p< 0.05, which demonstrated the accuracy of CCA analysis. As concluded from the regression model, the regression equation was: growth height = 142.728 − 1.514TC + 30.218AP − 5.083TN + 10.839AK + 1.234mc, and as shown in Table 7, AK also had great influence on density with its P< 0.05. Its regression equation was: density = −68.216 − 4.609TC + 9.566AP − 35.492TN + 19.779AK − 1.591mc.

**Table 6:** Correlation coefficient of the linear multivariate regression analysis (a).

Model	Unstandardized coefficients		Standardized coefficients	t	Sig.
	B	Std. Error	Beta		
Constant	142.728	28.532		5.002	0.001
TC	-1.514	1.808	-0.249	-0.837	0.422
AP	30.218	25.657	0.522	2.482	0.032
TN	-5.083	2.048	-0.296	-1.178	0.266
AK	10.839	3.945	0.655	2.748	0.021
mc	1.234	2.227	0.106	0.554	0.592

**Table 7:** Correlation coefficient of the linear multivariate regression analysis (b).

Model	Unstandardized coefficients		Standardized coefficients	t	Sig.
	B	Std. Error	Beta		
Constant	-68.216	52.876		-1.290	0.226
TC	-4.609	5.210	-0.172	-0.885	0.397
AP	9.566	10.817	0.223	0.884	0.397
TN	-35.492	81.192	-0.079	-0.437	0.671
AK	19.779	8.255	0.595	2.396	0.038
mc	-1.591	7.915	-0.031	-0.201	0.845

## Discussion

Most seeds have strong or weak dormancy characteristics. The germination of seeds is determined by external environmental and internal physiological factors [59]. The change of soil properties by the addition of substrate can improve the external environment to promote the germination of seeds, which will in turn affect the species and diversity. The highest diversity indexes were presented in experimental groups with mixed substrates (S2 Table), with its Shannon-Wiener ranging from 0.978–1.898, Simpson ranging from 0.418 to 0.703, Pielou ranging from 0.431 to 0.717. Compared with the highest values of these three indices in groups with single substrate, 1.225, 0.571, 0.695 respectively, the advantages of mixed substrates was shown. From Table 2, it’s evident that greater number of species was presented in groups with mixed substrate, ranging from 15-21.The dominant species of *Setaria viridis*、 *Digitaria sanguinalis* and *Cyperus compressus* have more seedlings than those with single substrates. Also the germination of *Chenopodium glaucum*、*Sonchus wightianus*、*Amaranthus cruentus* and *Chenopodium album* in groups with mixed substrate contributed a lot to enhance the diversity.

Testing Plot No. 16 had the highest diversity indexes with also the highest density of 14000 germinants/m^2^ and the highest species of 21. Also noteworthy is the fact that three arbor seedlings with the names of *Ailanthus altissima*、 *Fraxinus chinensis* and *Koelreuteria paniculata* have seeds with strong dormancy. Compared with single substrate, whether organic or inorganic substrate, the mixed substrates will improve soil condition quickly. It can provide enough nutrients、space and air flux to promote the germination of more seeds. In the practical application projects, considering the disadvantages of a too small or too large unit weight, poor ventilation or too much ventilation of a single substrate, the mixture of 2–3 substrates is more desirable[60]. This conclusion can be intuitively reflected in the CCA quadrats ordination graph, where mixed substrates composed of rice husk carbon and vermiculite could markedly improve the content of organic elements such as K、P. The reason being that rice husk carbon could significantly enhance available potassium in soil, increase the micronutrient levels in soil such as magnesium and manganese, while vermiculite, as a type of inorganic matrix, also contains a little K element. The improvement of K and P content in soil can also help to increase the diversity level of the community [61]. Mixed substrate composed of turfy soil and perlite was relatively poor in providing organic elements, but it has great advantage in maintaining moisture in soil, because turfy soil has a high ability to retain water with its soft texture and perlite could absorb water equal to 3–4 times of its weight, giving the mixture the advantage of maintaining moisture and air.

The CCA species ordination graph intuitively reflected the clustering regularity, which was important to find the preference to soil conditions of target species. The first cluster was distributed in the area with relatively high content of AK and AP. These plants including: *Convolvulus arvensis*、*Setaria viridis*、*Sonchus wightianus*、*Chenopodium album*、*Pharbitis nil*, etc, didn’t have high requirements on soil moisture content, which indicated these plants could adapt to a relatively dry environment, but they had a relatively a high requirement for fertile soil. The same conclusion was drawn from the above research in the Baxian Mountain research [62], in which these plants were also found distributed around the areas with relatively higher AP and AK. The second cluster was distributed in the area with relatively high soil moisture content. In factual biotope, the arbor trees of *Ailanthus altissima* and *Fraxinus chinensis* all preferred humid soil, and the *Digitaria sanguinalis* and *Youngia japonica* could grow on the side of a river or pond. The third cluster was distributed at the bottom left area, and the included plants could be able to widely adapt to all kinds of environments. In actual biotope, plants such as *Chenopodium glaucum* and *Cirsium arvense var*. *integrifolium* were widely distributed in various types of habitats. This graph can provide useful instructions for the restoration of target species, especially native species by providing specific soil environment preferences.

After the germination experiment, the advantages of mixed substrate were witnessed, including high density and high diversity indexes. Also the clustering regularity of species on soil factors gradients were found, and two kinds of mixed substrates were found with different function to improve soil condition. AP and AK were found to be the most important factor to promote the growth and density for SSBs. The main application values of these results can be divided into two aspects: 1、 According to the restoration of target species, especially native species, the suitable germinated soil condition will be created accordingly by adding specific mixed substrate to help the germination of target seeds. 2、 In order to enhance its restoration effects, such as density, diversity, community height, etc, the suitable mixed substrates can be chosen to promote the ecological restoration effect. Therefore this experiment will provide significant reference data for SSB engineering projects.

## Conclusion

The mixed substrates showed a greater advantage for SSBs than single substrates, both in improving the number of species、density and diversity indexes. For improving the three indexes, the mixed substrate composed of rice husk carbon and vermiculite with the volume ratio 1:1 was the best when mixed again with surface soil at the ratio of 30%. The mixed substrate of turfy soil and perlite could effectively enhance the soil moisture content, while the mixed substrate of rice husk carbon and vermiculite could improve the nutritive element content of soil. Plants also reflected obvious regularities of distribution on soil factor gradients. Three obvious cluster groups were presented, of which the first cluster was distributed at the places with a relatively higher content of AK and AP; the second cluster was distributed at the places with relatively higher soil moisture content; and the third cluster of plants didn’t have any obvious relationship with soil physical and chemical properties. Through CCA ordination analysis, AK and AP were considered the most important soil factors. By linear multivariate regression analysis, the correlation coefficients between the AK, AP and the growth height and density were relatively high and significant. The linear regression equation was also significant with P< 0.05, and the equations were: growth height = 142.728 − 1.514TC + 30.218AP − 5.083TN + 10.839AK + 1.234mc; density = 68.216 − 4.609TC + 9.566AP − 35.492TN + 19.779AK − 1.591mc.

## Supporting Information

S1 Table Soil properties for CCA and linear multivariate regression. (XLSX)

S2 Table Data of Shannon-Wiener index, Pielou index and Simpson index. (XLSX)

## References

[1] Bakker JP, Poschlod P, Strykstra RJ, Bekker RM, Thompson K. Seed banks and seed dispersal: important topics in restoration ecology. Acta Botanica Neerlandica 1996 12; 45(4):461–490.

[2] Whittle CA, Duchesne LC, Needham T. The importance of buried seeds and vegetative propagation in the development of post fire plant communities. Environmental Reviews 1997; 5:79–87. 10.1139/er-5-1-79

[3] Andreu MG, Hedman CW, Friedman MH, Andreu AG. Can Managers Bank on Seed Banks When Restoring *Pinus taeda L*. Plantations in Southwest Georgia? Restoration Ecology 2009 9; 17(5):586–596. 10.1111/j.1526-100X.2008.00457.x

[4] Maranon T. Soil seed bank and community dynamics in an annual-dominated Mediterranean salt-marsh. Journal of Vegetation Science 1998; 9:371–378. 10.2307/3237101

[5] Ma MJ, Zhou XH, Du GZ. Effects of disturbance intensity on seasonal dynamics of alpine meadow soil seed banks on the Tibetan Plateau. Plant Soil 2013; 369:283–295. 10.1007/s11104-012-1560-5

[6] Lyaruu HVM, Backeus I. Soil seed bank and regeneration potential on eroded hill slopes in the KondoaIrangi Hills, central Tanzania. Applied Vegetation Science 2009; 2:209–214. 10.2307/1478984

[7] Keddy PA, Reznicek AA. The role of seed banks in the persistence of Ontario’s coastal plain flor. American Journal of Botany 1982; 69(1):13–22. 10.2307/2442827

[8] Marks PL, Mohler CL. Succession after elimination of buried seeds from a recently plowed field. Bulletin of the Torrey Botancical Club 1985; 112(4):376–382. 10.2307/2996038

[9] Nishihiro J, Nishihiro MA, Washitani I. Restoration of wetland vegetation using soil seed banks: lessons from a project in Lake Kasumigaura, Japan. Landscape and Ecological Engineering 2006; 2(2):171–176. 10.1007/s11355-006-0005-9

[10] Koh J, Ueda T, Morimoto Y. Evaluation of using soil seed bank and commercial seeds in nature revegetation. Journal of the Japanese Society of Revegetation Technology 2006; 32:62–67.

[11] Jimmon T, Sato H, Morimoto Y. A preliminary study of revegetation of disturbed area forest seed banks. Journal of the Japanese Society of Revegetation Technology 2000; 25:397–402.

[12] Hosogi D, Yonemura S, Kameyama A. Thickness of topsoil setting and effectiveness of fertilizer quantity and malting on banked slope revegetation with forest topsoil. Journal of the Japanese Society of Revegetation Technology 2006; 31:385–390.

[13] Yamabe S, Watanabe T. Effect of restoration of the natural environment by transplanting topsoil in the forest. Journal of the Japanese Society of Revegetation Technology 2004; 29:393–395. 10.7211/jjsrt.29.393

[14] Umehara T, Nagano M. Regeneration of forest at a dam site using stored surface soil seed banks. The Ecological Society of Japan 1997; 2:9–26.

[15] Nakamura K, Honda K, Taniguchi S. A case study on the revegetation method using topsoil seedbank in Yakushima Island. Journal of the Japanese Society of Revegetation Technology 2006; 32:203–206.

[16] Li HY, Sato H, Morimoto Y, Zhu L. Application of soil seed bank in wasteland revegetation project. Journal of Liaoning Technical University 2007; 26(1):140–142.

[17] Hassan B, Ahmad M, Mehrdad J. Effect of date-palm and perlite substrates on nutrients content and quality of tomato grown in soilless culture. Research On Crops 2012;13(1):258–261.

[18] Li HY, Sato H, Zhu L, Morimoto Y. The relationship analysis of plant species of soil seed bank and the soil vegetation in the collecting field. Ecology and Environment 2006; 15(4):791–795. 10.3969/j.issn.1674-5906.2006.04.027

[19] Bai WJ, Zhang JE, Quan GM. Hot Topics and Developing Trends in Soil Seed Bank. Soils 2012 4; 44(4):562–569.

[20] Li HY, Mo XQ, Hao C. A review of study on soil seed bank in the past thirty years. Ecology and Environmental Sciences 2009; 18(2): 731–737. 10.3969/j.issn.1674-5906.2009.02.060

[21] Wang J, Bai Y. The hot topics and perspectives of soil seed bank research. Ecology and Environment 2006; 15(6):1372–1379. 10.3969/j.issn.1674-5906.2006.06.047

[22] Liang YY, Li HY, Mo XQ, Ma CH. Topsoil application in vegetation restoration in Japan.Chinese Journal of Applied Ecology 2009; 20(11):2832–2838. 20136024

[23] Mo XQ. Soil seed bank and its application in vegetation restoration in coastal area. D.Sc. Thesis: Nankai University.2012.

[24] Page MJ, Baxter GS, Lisle AT. Evaluating the adequacy of sampling germinable soil seed in semi-arid systems. Journal of Arid Environments 2006; 64(2):323–341. 10.1016/j.jaridenv.2005.05.003

[25] Zhang DJ, Zhang J, Yang WQ. Wu FZ, Huang YM. Plant and soil seedbankdiversityacross a range of ages of Eucalyptus grandis plantations afforested on arable lands. Plant and Soil 2014; 376(1–2):307–325. 10.1007/s11104-013-1954-z

[26] Carlile WR. The effects of the environment lobby on the selection and use of growing media. Acta Hort 1999; 481:587–596. 10.17660/ActaHortic.1999.481.69

[27] Gruda N, Schnitzler WH. Suitability of wood fiber substrates for production of vegetable transplants II. Scientia Horticulturae 2004; 100(1–4):333–340. 10.1016/j.scienta.2003.10.001

[28] Kang HM, Zhang QX, Tang J. Research Advances on Growth Media. Chinese Journal of Soil Science 2005; 36(1):124–127.

[29] Carmona E, Moreno MT, Avilés M, Ordovas J. Composting of wine industry wastes and their use as a substrate for growing soilless ornamental plants. Spanish Journal of Agricultural Research 2012; 10(2):482–491. 10.5424/sjar/2012102-320-11

[30] Caron J, Nkongolo LM. Aeration in growing media: recent development. Acta Hort 1999; 481: 545–551. 10.17660/ActaHortic.1999.481.64

[31] Sun SR, Yang QS, Dong XY, et al Analysis of mineral element contents and physical and chemical properties of corn stalk substrate. Transactions of the CSAE 2008; 24(6):41–44.

[32] Shahidul Islam Md, Khan S, Ito T, Maruo T, Shinohara Y.Characterization of the physico-chemical properties of environmentally friendly organic substrates in relation to rockwool.Hort.Sci.&Biotech. 2002; 77(2):143–148.

[33] He MX. The feature analysis of soil seed bank by adding substrates. M.Sc. Thesis: Nankai University.2015.

[34] Hill MO. Canonical correspondence analysis: A new multivariate method. Journal of the Royal Statistical Society 1974; 23:340–354.

[35] Ter Braak CJF. Canonical correspondence analysis: A new eigenvector technique method for multivariate direct gradient analysis. 1986; 67: 1167–1179. 10/1986; 67(5):1167–1179

[36] Cao J, Miao YM, Feng F, Xu Q, Zhang QD, Bi RC. Comparison of different treatments of rare species in canonical correspondence analysis. Chinese Journal of Plant Ecology 2015; 39(2):167–175. 10.17521/cjpe.2015.0016

[37] Mohsen BB, Salehia MH, José AMC, Mohammadia J, Toomanianc N, Borujenid IE. Using Canonical Correspondence Analysis (CCA) to identify the most important DEM attributes for digital soil mapping applications. Catena 2011; 86(1):66–74. 10.1016/j.catena.2011.02.009

[38] Bhosale MD, Singh TP.Comparative study of feed-forward neuro-computing withmultiplelinearregressionmodel for milk yield prediction in dairy cattle. Current Science 2015 6; 108(12):2257–2261.

[39] Landman GB, Kolka RK, Sharitz RR. Soil seed bank analysis of planted and naturally revegetating thermally-disturbed riparian wetland forests. Wetlands 2007; 27:211–223. 10.1672/0277-5212(2007)27[211:SSBAOP]2.0.CO;2

[40] Hager HA, Rupert R, Quinn LD. Escaped Miscanthus sacchariflorus reduces the richness and diversityof vegetation and the soilseed bank. Biological Invasions 2015; 17(6):1833–1847. 10.1007/s10530-014-0839-2

[41] Hanin N, Quaye M, Westberg E, Barazani O. Soilseedbankand among-years geneticdiversityin arid populations of Eruca sativa Miller (Brassicaceae).Journal of Arid Environments 2013; 91:151–154. 10.1016/j.jaridenv.2013.01.004

[42] Zhang DJ, Zhang J, Yang WQ. Wu FZ, Huang YM. Plant andsoilseed bank diversity across a range of ages of Eucalyptus grandis plantations afforested on arable lands. Plant and Soil 2014; 376(1–2):307–325. 10.1007/s11104-013-1954-z

[43] Srivastava R, Singh KP. Diversity in weed seed production and the soil seed bank: Contrasting responses between two agroecosystems. Weed Biology and Management 2014;14(1):21–30. 10.1111/wbm.12029

[44] Heydari M, Hassan P, Omid E. Germination characteristics and diversity of soil seed banks and above-ground vegetation in disturbed and undisturbed oak forests. Forest Science and Practice 2013;15(4):286–301. 10.1007/s11632-013-0413-5

[45] Davies RJP, Whalen MA, Mackay DA. Does soil seedbankdiversitylimit post-fire regeneration in small, fragmented, long-unburnt remnants of fire adapted vegetation. Biological Conservation 2013; 158:287–295. 10.1016/j.biocon.2012.08.013

[46] Mesquita R, Luiz M, Andrade D, Alves L, Esfrain PW. Floristicdiversityof thesoilweedseedbankin a rice-growing area of Brazil in situ and ex situ evaluation. Acta Botanica Brasilica 2013; 27(3):465–471.

[47] Jin ZJ, Li ZY, Li Q, Hu QJ, Yang RM, Tang HF, et al Canonical correspondence analysis of soil heavy metal pollution, microflora and enzyme activities in the Pb-Zn mine tailing dam collapse area of Sidi village, SW China. Environmental Earth Sciences 2015; 73(1):267–274. 10.1007/s12665-014-3421-4

[48] Zhao ZH, Jiang Y, Xia LL, Mi TF, Yan WM, Gao YZ, et al Application of canonical correspondence analysis to determine the ecological contribution of phytoplankton to PCBs bioaccumulation in Qinhuai River, Nanjing, China. Environmental science and pollution research 2014; 21:3091–3103. 10.1007/s11356-013-2265-x 24197969

[49] Shang ZH, Deng B, Ding LM, Ren GH, Xin GS, Liu ZY, et al The effects of three years of fencing enclosure on soil seed banks and the relationship with above-ground vegetation of degraded alpine grasslands of the Tibetan plateau. Plant Soil 2013; 364(1–2):229–244. 10.1007/s11104-012-1362-9

[50] Zhai FQ, Xu N, Mo XQ. Characteristics of soil seed banks and soil physical and chemical properties of hydro-fluctuation belt in Jiyun Canal. Research of Environmental Sciences 2013; 26(1): 97–102.

[51] Hao C, Liang YY, Meng WQ, Li HY, Ma CH. Relations between plant community characteristic and soil physicochemical factors in natural wetlands of Binhai New District, Tianjin.Wetland Science 2009;7(3):266–272.

[52] Liang J, Hua SS, Zeng GM, Yuan YG, Lai X, Li XD, et al Application of weight method based on canonical correspondence analysis for assessment of Anatidae habitat suitability: A case study in East Dongting Lake, Middle China. Ecological Engineering 2015; 77: 119–126. 10.1016/j.ecoleng.2015.01.016

[53] Borowiak K, Jusik S, Zbierska J.Canonical Correspondence analysis (CCA) as a tool for interpretation of bioindication plants response to ambient air pollution. Fresenius Environmental Bulletin 2011; 20(9):2264–2270.

[54] Zhang Z, Coillie FV, Ou XK, Wulf DR. Integration of Satellite Imagery, Topography and Human Disturbance Factors Based on Canonical Correspondence Analysis Ordination for Mountain Vegetation Mapping: A Case Study in Yunnan, China. Remote Sensing 2014; 6:1026–1056. 10.3390/rs6021026

[55] Li SX, Wang ZH, Malhi SS, Li SQ, GAO YJ, Tian XH. Nutrient and water management effects on crop production, and nutrient and water use efficiency in dry land areas of China. Advances in Agronomy 2009; 102:223–265. 10.1016/S0065-2113(09)01007-4

[56] Tai NH, Park JS, Ahn TI, Lee JH, Myoung DJ, Cho YY, et al Analysis of relationship among growth, environmental factors and transpiration in soilless culture of paprika plants. Korean Journal of Horticultural Science& Technology 2010; 28:59–64.

[57] Moreira DR, Cardoso VJM. Effect of soil moisture content and the irrigation frequency on the sugarcane germination. Pesqui Agropecu Bras 1998; 33:721–729.

[58] Badwaik LS, Prasad K, Deka SC. Optimization of extraction conditions by response surface methodology for preparing partially defatted peanut. International Food Research Journal.2012; 19(1):341–346.

[59] Fisher JL, Veneklaas EJ, Loneragan WA and Dixon KW. Soil seed bank compositional change constrains biodiversity in a species-rich invaded woodland, Biological Conservation 2009; 142: 256–269. 10.1016/j.biocon.2008.10.019

[60] Jong HS, Jung ES. Changes in electrical conductivity and moisture content of substrate and their subsequent effects on transpiration rate, water use efficiency and plant growth in the soilless culture of paprika (*Capsicum annuum L*.) Horticulture Environment and Biotechnology 2015;56(2):178–185. 10.1007/s13580-015-0154-6

[61] Hong JM, Liu S, Shi GP. Soilseedbanktechniques for restoring wetland vegetationdiversityin Yeyahu Wetland, Beijing. Ecological Engineering 2012; 42:192–202. 10.1016/j.ecoleng.2012.01.004

[62] He MX, Mo XQ, Li HY. Analysis of soil seed bank characteristics in Baxian mountain of Jixian county based on detrended canonical correspondence analysis. Bulletin of Soil and Water Conservation 2015; 35(3):325–330.

